# A systematic review on experimental studies about patient adherence to treatment

**DOI:** 10.1002/prp2.1166

**Published:** 2024-01-11

**Authors:** Frans Folkvord, Ana Roca‐Umbert Würth, Karlijn van Houten, Aad R. Liefveld, Jim Ingebretsen Carlson, Nadine Bol, Emiel Krahmer, Gwenn Beets, Rachel Drbohlav Ollerton, Eva Turk, Harald Hrubos‐Strøm, Hamza Nahoui, Gunnar Einvik, Henrik Schirmer, Anne Moen, Jaime Barrio‐Cortes, Beatriz Merino‐Barbancho, Peña Arroyo, Giuseppe Fico, Luís Midão, Rute Sampaio, João A. Fonseca, Katja Geipel, Kathrin Scheckenbach, Laura E. de Ruiter, Francisco Lupiáñez‐Villanueva

**Affiliations:** ^1^ PredictBy Barcelona Spain; ^2^ Tilburg Center for Cognition and Communication (TiCC), Department of Communication and Cognition Tilburg University Tilburg The Netherlands; ^3^ Link2Trials Hilversum The Netherlands; ^4^ Institute for Health and Society University of Oslo Oslo Norway; ^5^ Akershus University Hospital Lørenskog Norway; ^6^ Institute of Clinical Medicine University of Oslo Oslo Norway; ^7^ Foundation for Biosanitary Research and Innovation in Primary Care Madrid Spain; ^8^ Research Unit, Primary Healthcare Management, Madrid Health Service Madrid Spain; ^9^ University Camilo José Cela Madrid Spain; ^10^ Universidad Politécnica de Madrid, Life Supporting Technologies Research Group Madrid Spain; ^11^ Associate Laboratory i4HB – Institute for Health and Bioeconomy, UCIBIO – Applied Molecular Biosciences Unit, Porto4Ageing – Competence Centre on Active and Healthy Ageing, Faculty of Pharmacy of the University of Porto Porto Portugal; ^12^ CINTESIS@RISE, Department of Biomedicine Faculty of Medicine of the University of Porto Porto Portugal; ^13^ MEDIDA, Medicina, EDucação, I&D e Avaliação Lda Portugal; ^14^ MEDCIDS, Department of Community Medicine, Information and Health Decision Sciences, Faculty of Medicine University of Porto Porto Portugal; ^15^ Department of Otorhinolaryngology Heinrich‐Heine‐University Düsseldorf Germany; ^16^ Behavioral Science Consortium, Astellas Pharma Northbrook Illinois USA; ^17^ Universidad Oberta de Catalunya, Information and Communication Sciences Barcelona Spain

**Keywords:** dimensions, experimental studies, factors, medication, patient adherence, systematic review

## Abstract

A better understanding of patients' adherence to treatment is a prerequisite to maximize the benefit of healthcare provision for patients, reduce treatment costs, and is a key factor in a variety of subsequent health outcomes. We aim to understand the state of the art of scientific evidence about which factors influence patients' adherence to treatment. A systematic literature review was conducted using PRISMA guidelines in five separate electronic databases of scientific publications: PubMed, PsycINFO (ProQuest), Cochrane library (Ovid), Google Scholar, and Web of Science. The search focused on literature reporting the significance of factors in adherence to treatment between 2011 and 2021, including only experimental studies (e.g., randomized controlled trials [RCT], clinical trials, etc.). We included 47 experimental studies. The results of the systematic review (SR) are grouped according to predetermined categories of the World Health Organization (WHO): socioeconomic, treatment, condition, personal, and healthcare‐related factors. This review gives an actual overview of evidence‐based studies on adherence and analyzed the significance of factors defined by the WHO classification. By showing the strength of certain factors in several independent studies and concomitantly uncovering gaps in research, these insights could serve as a basis for the design of future adherence studies and models.

## Graphical abstract

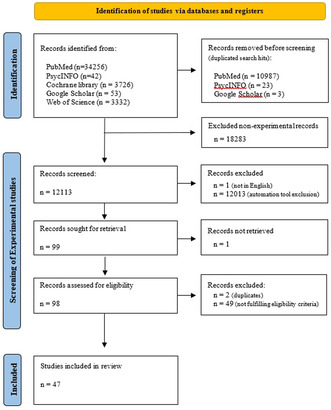

**Abbreviations**
EU: European Union
HCP: Healthcare professional
ML: machine learning
N: Number
PAP: positive airway pressure
PRISMA: Preferred Reporting Items for Systematic Reviews and Meta‐Analyses
RCT: Randomized controlled trials
SR: systematic review
US: United States
WHO: World Health Organization

## 1 INTRODUCTION

Patients' adherence to treatment is important to maximize the benefit of healthcare provided to patients and is a key factor in a variety of subsequent health outcomes. We understand adherence to treatment as the process in which the patient engages in a health, technology, or medication treatment that was agreed upon together with a healthcare professional. Adherence includes meeting the following conditions that are relevant to the treatment: (1) taking prescribed medication correctly at the minimum clinical threshold agreed upon, including initiation, dosage, and persistence; (2) carrying out recommended health behaviors, such as attending follow‐up appointments and/or implementing lifestyle changes (e.g., avoiding certain foods or engaging in specific exercise), at the minimum clinical threshold agreed upon.

Currently, lack of adherence is associated with personal suffering, poorer health outcomes, and a significant burden on healthcare costs/budgets.^1^ Overall, up to 125 000 premature deaths per year in the US^2^ and 200 000 in the EU^3^ can be related to non‐adherence. On average, 25 percent of patients do not engage in recommendations for prevention and disease management activities, including medication intake, technical treatment modalities (e.g., positive airway pressure [PAP]), appointment scheduling, screening, exercise, and dietary changes.^4^, ^5^ More general estimates show that almost 50 percent of patients do not adhere to treatment recommendations.^6^ When preventive or treatment regimens are complex and/or require lifestyle changes and modification of existing habits, non‐adherence can be as high as 70 percent.^5^, ^7^ Treatment non‐adherence has been identified as a major barrier to the effective (self‐)management of chronic conditions, leading to poorer health outcomes among patients, higher hospitalization rates, and increased mortality. Therefore, non‐adherence eventually causes an additional financial burden on healthcare systems and the overall social costs.^1^, ^8^

Given the proportion of the patient population that does not adhere to treatments, efforts to improve treatment adherence represent a great opportunity to enjoy the full benefit of treatment and enhance health outcomes while ensuring quality, efficiency, and sustainability of the healthcare system. Action to better understand the complexity of factors that influence patients' capacities and the reasons driving behavior change toward treatment adherence, including heterogeneity of treatments, is urgently needed to address the situation, focusing on “*real individuals*” instead of the “*ideal individuals*”.^9^ Therefore, for effective care provision, it is necessary to activate the patient and the patient's community of support to better understand the complexity of factors and improve adherence to treatment. The main aim of this systematic review (SR) is to understand the state of the art of scientific evidence about the relationship and impact of different types of interventions developed to increase adherence to treatment.

## 2 METHODS

The information sources consulted for this SR were the following electronic databases of scientific publications: PubMed, PsycINFO (ProQuest), Cochrane library (Ovid), Google Scholar, and Web of Science.

### 2.1 Search strategy

Table 1 represents the basic search string developed for this SR. The full list of search strings that were used to inspect and search each of the databases mentioned above is available in Supplementary Material. The search strings were developed through several discussions among all the authors and were pretested several times in different databases in order to make sure valid and reliable outcomes were obtained.

**TABLE 1:** Basic search string developed for this systematic review.

**Search String**
(Treatment Adherence and Compliance[mh] OR Patient Compliance[mh] OR Patient Dropouts[mh] OR Therapeutic Adherence[mh] OR Therapeutic Adherence and Compliance[mh] OR Treatment Adherence[mh] OR Non‐Adherent Patient[mh] OR Patient Adherence[mh] OR Patient Non‐Adherence[mh]) AND (Following treatment [tiab] OR Following therapy [tiab] OR Following medication [tiab] OR Adhere [tiab] OR Adherence [tiab] OR Nonadherence [tiab] OR Compliance [tiab] OR Noncompliance [tiab] OR Concordance [tiab] OR Adherent [tiab] OR Nonadherent [tiab] OR Compliant [tiab] OR Noncompliant [tiab] OR Concordant [tiab] OR Patient dropouts [tiab] OR Treatment refusal [tiab] OR Therapy refusal [tiab] OR Medication refusal [tiab] OR Directly observed therapy [tiab] OR Behavior change [tiab] OR Persistence [tiab] OR Nonpersistence [tiab] OR Discontinuation [tiab] OR Burden of treatment [tiab] OR Treatment inertia [tiab] OR Medication possession ratio [tiab] OR Proportion of days covered [tiab] OR PAP treatment adherence [tiab] OR Positive airway pressure treatment adherence [tiab] OR Adherence to lifestyle changes [tiab] OR dietary adherence [tiab]) AND (Factor [tiab] OR Factors [tiab] OR Dimension [tiab] OR Dimensions [tiab] OR Models [tiab] OR Variable* [tiab] OR Predict* [tiab] OR Modifier* [tiab] OR Influenc* [tiab] OR Determin* [tiab] OR Associat* [tiab] OR Indicat* [tiab] OR Facilitat* [tiab] OR Risk factor [tiab] OR Barrier [tiab] OR Barriers [tiab]) AND (English[la])

### 2.2 Eligibility criteria

The focus of the SR was to analyze the literature reporting on the effect of factors in adherence to treatment. The initial review included both experimental and non‐experimental studies, and the results of both searches were analyzed independently. The SR reported here focused only on the experimental studies, excluding the non‐experimental ones that will be analyzed and reported in a separate article. Studies published within the last ten years (2011–2022) were considered. All eligible studies had to be written in English. The population of interest in the studies under review was restricted to adult human patients who had been or were planning to be under treatment for a certain chronic or acute physical condition. Consequently, treatment was defined as not only medication taking but also engaging in other health behaviors, such as attending follow‐up appointments, implementing lifestyle changes (e.g., avoiding certain foods, engaging in specific exercise), and using medical devices. Finally, eligible publications had to report the effect of one or more factors on treatment adherence to be included in this review. The inclusion and exclusion criteria for the studies eligible for this review are summarized in Table 2.

**TABLE 2:** Summary of inclusion and exclusion criteria.

Evidence	Publications of studies that assess the effect of one or more factors on treatment adherence.
Publication characteristics	Peer reviewed articles published in English within the last ten years (2012–2022)
Population	Studies considering adult human participants (≥ 16 years old). For reviews and overviews, only those including ≥80% of included studies analyzing adult population
Condition type	Both, chronic and acute physical conditions. Studies focused on patients suffering from mental health disorders were excluded from the analysis
Treatment type	The studies eligible for this review were those that analyze adherence to any kind of treatment or medical recommendation, meaning not only medication taking but also other health behaviors, such as attending follow‐up appointments, implementing lifestyle changes (e.g., avoiding certain foods, engaging in specific exercise), and using medical devices
Data included	Studies that at least report, for the analyzed factors, the direction of the effect and its statistical significance

### 2.3 Selection of studies for inclusion

#### 2.3.1 Data management

Data were managed using Microsoft Excel and plain text files stored in Microsoft SharePoint for easy access. The search hits (including publication title, authors, abstract, and DOI) were downloaded in .csv, .txt, or .xlsx format, depending on the database options. A file containing all hits for each search was stored in Microsoft's SharePoint. Search hits from different databases were merged, duplications were removed, and non‐experimental records were excluded, resulting in one file prepared for the screening of the search hits for experimental studies only (*n* = 12 113).

#### 2.3.2 Selection process

**FIGURE 1:**
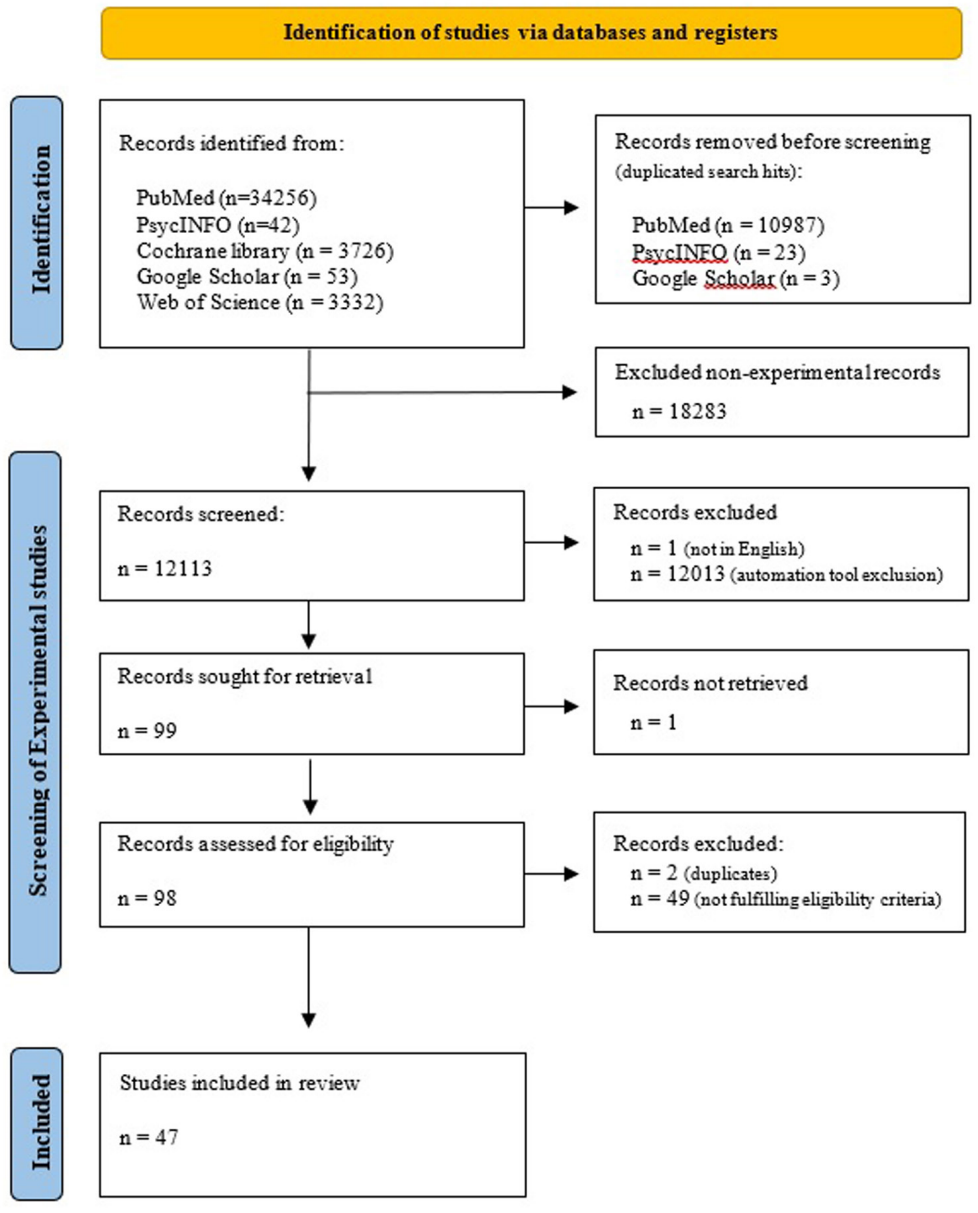
PRISMA flow diagram. Diagram adapted from Page et al. (2021).

The outcome of the study was screened and selected using an open‐source machine learning (ML)‐aided pipeline applying active learning: ASReview, Active learning for Systematic Reviews.^10^ ASReview is a tool that increases the efficiency of screening titles and abstracts by determining prioritization with active learning. The ASReview tool is extensively tested and validated and has shown to achieve better performance in SR's than manually evaluation titles and abstracts.^10^ The tool was initially trained for the current study with ten relevant and ten irrelevant publications selected by two independent researchers (ARU & KvH). After feeding the tool with the training publications, the tool returned the set of hits ordered according to relevance priority. These results were checked by the same two independent researchers. In case of several irrelevant results among the top priority hits, the tool was further trained by manually screening at least 1% of the total number of publications in the whole set. Publications selected for further full‐text review (*n* = 99) were those prioritized by ASReview. For each assigned publication, authors checked each criterion and assessed the inclusion of only those publications that met all criteria. Each publication was reviewed by a second independent author following concordant and stratified criteria. The full list of studies included for full‐text review as well as the inclusion and exclusion criteria can be consulted in Supplementary Material. For the selected publications (*n* = 47), authors annotated some additional publication details (e.g., country of the study, participants included, disease area, factors affecting adherence considered, study design, type of experimental design, etc.). The total number of records after each screening round was documented using the PRISMA flow diagram template (see Figure 1).

Subsequently, the data related to the effect of interventions to increase adherence were extracted from each study. These effects were grouped according to the following dimensions: socioeconomic, treatment, condition, personal, and healthcare‐related factors, which were based on WHO's five dimensions of treatment adherence.^11^ For each adherence factor, both the inclusion and exclusion in each of the reviewed studies were reported, as well as evidence of a significant association of that specific factor with treatment adherence. Variables related to the characteristics of the study, study sample, and study intervention were also extracted.

## 3 RESULTS

The included studies are grouped according to the following categories: socioeconomic, treatment, condition, personal, and healthcare‐related factors, based on the WHO dimensions of patients' adherence to treatment.

### 3.1 Socioeconomic factors

Socioeconomic factors can be divided into those factors related to social or environmental variables, economic factors, and those related to the lifestyle of patients. Among the studies analyzed, we identified four studies that showed a significant effect of social or environmental factors (i.e., social interaction and support networks) on treatment adherence.^12^, ^13^, ^14^, ^15^ Concerning the set of economic factors, several studies reported a significant association between adherence to treatment and financial status,^16^, ^17^, ^18^, ^19^ education and literacy,^14^, ^16^, ^20^ employment,^15^, ^16^, ^21^ and living condition of patients.^19^, ^22^, ^23^ This SR also identified scientific evidence on the effect of patients' lifestyle on treatment adherence. The lifestyle factors with a reported significant effect are substance use and abuse^21^, ^22^, ^24^ and physical activity.^18^, ^25^ Among the studies reviewed, no reference was made to the study of the effect of the social situation of the patient in adherence to treatment. For full reference to the data extracted, see Table 3.

**TABLE 3:** Reported evidence on the effect of socioeconomic factors on treatment adherence.

Socioeconomic	Exp. studies evaluating the factor	Experimental studies reporting significant effects
*Social or environmental factors*		
Social interaction and support networks	9	*n* = 4^12^, ^13^, ^14^, ^15^
Social stigma of a condition, socioeconomic status	1	No significant effects reported
Access to treatment center, culture and lay beliefs about illness and treatment	4	No significant effects reported
Health‐related media use (e.g., searching for information)	1	No significant effects reported
*Economic factors*		
Financial status	9	*n* = 4^16^, ^17^, ^18^, ^19^
Education and literacy	18	*n* = 3^14^, ^16^, ^20^
Employment	11	*n* = 3^15^, ^16^, ^21^
Living condition	5	*n* = 3^19^, ^22^, ^23^
Insurance access and coverage	3	No significant effects
*Patients' lifestyle factors*		
Substance (ab)use (Including smoking and alcohol)	10	*n* = 3^21^, ^22^, ^24^
Physical activities	3	*n* = 2^18^, ^25^

*Note*: Total number of experimental studies = 47.

### 3.2 Factors related to the healthcare system

The healthcare system‐related factors were divided into two sets of factors: those concerning the relationship between the patient and the healthcare professional (HCP), and those directly related to the healthcare system. In this SR, ample evidence showed that provision of patient education, training, and follow‐up of the patient by the HCP significantly increased adherence.^23^, ^26^, ^27^, ^28^, ^29^, ^30^, ^31^, ^32^, ^33^, ^34^, ^35^ Moreover, the patients' trust in their HCP^17^ and HCPs' time available for consultation^36^ were also found to have a significant effect on patients' adherence to treatment. When considering only the healthcare system‐related factors, it was found that both the provision of feedback and training to the HCP and the support of the community influence patients' adherence to treatment.^37^, ^38^ Among the studies reviewed, no reference was made to the study of the effect of the “quality and cost of health services”, “Provider continuity”, “Regulation process”, or “Drug supply” in adherence to treatment. For a complete reference to the data extracted, see Table 4.

**TABLE 4:** Reported evidence on the effect of healthcare‐related factors on treatment adherence.

Healthcare‐related factors	Exp. studies evaluating the factor	Experimental studies reporting significant effects
*Related to the patient – HCP relationship*		
Relationship with HCP	3	No significant effect reported
Communication abilities	2	No significant effect reported
Trust in provider	1	*n* = 1^17^
Provision of patient education, training and follow‐up	21	*n* = 11^23^, ^26^, ^27^, ^28^, ^29^, ^30^, ^31^, ^32^, ^33^, ^34^, ^35^
Time availability of consultation (Incl. Frequency of visits)	4	*n* = 1^36^
*Directly related to the healthcare system*		
Access or barriers to the system	2	No significant effect reported
Insurance coverage and co‐payment	2	No significant effect reported
Provision of feedback and training to HCPs	3	*n* = 1^37^
Community support available to patients	2	*n* = 1^38^

*Note*: Total number of experimental studies = 47.

### 3.3 Disease‐related factors

The third dimension of adherence considered in this SR concerned disease‐related factors. Two studies found evidence for the effect of progress, duration, and severity of the disease and its symptomatology as an influencer of adherence.^20^, ^39^ Furthermore, several studies identified the existence of co‐morbidities as a factor significantly affecting adherence to treatment.^12^, ^18^, ^37^ In addition, the level of disability caused by the condition at the physical, psychological, social, and vocational levels has also been found to play a significant role in the level of patients' adherence to treatment, according to three articles.^16^, ^25^, ^40^ For full reference to the data extracted, see Table 5.

**TABLE 5:** Reported evidence on the effect of Condition or disease‐related factors on treatment adherence.

Disease‐related factors	Exp. studies evaluating the factor	Experimental studies reporting significant effects
Progress, duration, and severity of the condition and its symptomatology	8	*n* = 2^20^, ^39^
Level of disability caused by the condition at the physical, psychological, social, and vocational levels	6	*n* = 3^16^, ^25^, ^40^
Existence of co‐morbidities (including depression)	15	*n* = 3^12^, ^18^, ^37^

*Note*: Total number of experimental studies = 47.

### 3.4 Treatment‐related factors

Several adherence factors associated with patients' treatment have also been identified as modifiers of adherence. These treatment‐related factors can be further categorized as factors related to the treatment regimen, the effects of the treatment, and the treatment properties. Regarding the treatment regimen, ten articles in this SR reported scientific evidence that complexity and duration of the treatment have a causal effect on patients' adherence levels.^19^, ^21^, ^24^, ^33^, ^41^, ^42^, ^43^, ^44^, ^45^, ^46^ Similarly, another study identified how the treatment properties, specifically the formulation and physical properties of the medication, had a significant effect on the patients' adherence levels.^30^ Focusing on the treatment effects, only one article found evidence that the appearance of beneficial effects or side effects and experience of failures in previous treatments to influence adherence.^18^, ^22^ Among the studies reviewed, no reference was made to the study of the effect of the “Interference in the routine of the patient” or “Cost of treatment” in adherence to treatment. Furthermore, what is missing is the heterogeneity of treatment effect at local level. For full reference to the data extracted, see Table 6.

**TABLE 6:** Reported evidence on the effect of treatment‐related factors on treatment adherence.

Treatment‐related factors	Exp. studies evaluating the factor	Experimental studies reporting significant effects
*Treatment regimen*		
Complexity and duration of the treatment (including dosing regimen, tooling, and amount of medicines taken & irregularity)	19	*n* = 10^19^, ^21^, ^24^, ^33^, ^41^, ^42^, ^43^, ^44^, ^45^, ^46^
Patient friendliness of the regimen	3	No significant effect reported
Variation and changes of the treatment	2	No significant effect reported
*Treatment effects*		
Appearance of the beneficial effects or side effects (Treatment beliefs)	5	*n* = 1^18^
Experience of failures in previous treatments	3	*n* = 1^22^
*Treatment properties*		
Formulation and physical properties of the medication	3	*n* = 1^30^

*Note*: Total number of experimental studies = 47.

### 3.5 Patient‐related factors

The final dimension in the WHO framework is patient‐related factors, which was further divided into three sets of factors: unalterable characteristics, cognitive and psychological factors, and behavioral factors. Regarding the first factor, ample studies in this SR identified demographics to play a significant role in adherence to treatment,^16^, ^18^, ^20^, ^21^, ^24^, ^37^, ^40^, ^41^, ^45^, ^46^, ^47^, ^48^ while only one study showed this significance for experience with treatment and treatment setting.^49^ Also, the physical characteristics of the patients were found to be predictors for adherence to treatment in this SR.^16^, ^22^ Among the cognitive and psychological factors that were studied, health literacy,^16^ perceptions, beliefs, and concerns of the patients regarding their condition,^16^, ^39^, ^47^ patients' knowledge about their treatment,^18^, ^20^, ^49^ and patients' knowledge about their disease^14^, ^24^, ^39^, ^49^ were reported as predictors for adherence to treatment. Finally, some behavioral factors were found to have an effect on adherence, such as the lifestyle of the patient^18^, ^24^, ^43^, ^50^ and the planning abilities and self‐efficacy, which were found in three studies.^24^, ^44^, ^47^ The table shows the results of the quantification of the factors affecting adherence based on the SR of experimental studies. For full reference to the data extracted, see Table 7.

**TABLE 7:** Reported evidence on the effect of patient‐related factors on treatment adherence.

Patient‐related factors	Exp. studies evaluating the factor	Experimental studies reporting significant effects
*Unalterable characteristics*		
Demographics	26	*n* = 12^16^, ^18^, ^20^, ^21^, ^24^, ^37^, ^40^, ^41^, ^45^, ^46^, ^47^, ^48^
Experience with treatment and treatment setting	2	*n* = 1^49^
Physical characteristics of the patients (including clinical features (BP, pulse, hematocrit))	12	*n* = 2^16^, ^22^
*Cognitive and psychological factors*		
Health literacy	4	*n* = 1^16^
Perceptions, beliefs, and concerns of the patients regarding their condition	8	*n* = 3^16^, ^39^, ^47^
Motivation and ability to manage the condition	5	No significant effect reported
Patients' knowledge about the treatment	7	*n* = 3^18^, ^20^, ^49^
Patients' knowledge about the disease	8	*n* = 4^14^, ^24^, ^39^, ^49^
*Behavioral factors*		
Lifestyle of the patients	9	*n* = 5^18^, ^24^, ^34^, ^43^, ^50^
Organization	2	No significant effect reported
Planning abilities	9	*n* = 3^24^, ^44^, ^47^

*Note*: Total number of experimental studies = 47.

### 3.6 Inclusion of covariates

In total, 7 of the 47 studies analyzed for this SR reported having controlled the effect of any covariate. From those studies, the table presents which factors these studies analyzed as covariates. As Table 6 shows, demographic factors are the most used as covariates in the reviewed studies. These are followed by factors related to the characteristics of the treatment or disease or the economic situation of the patient. Other factors are rarely analyzed as covariates in the reviewed studies (Table 8).

**TABLE 8:** Inclusion of covariates in studies analyzing the effect or association of diverse factors and the level of treatment adherence.

Covariates considered	# Papers (*N* total papers = 47)
*Socioeconomic*	
Social or environmental factors [(a) social interaction and support networks; (b) social stigma of a condition, socioeconomic status; (c) access to treatment center, culture and lay beliefs about illness and treatment; (d) health‐related media use	*n* = 1^12^
Economic factors [(a) financial status; (b) education and literacy; (c) employment; (d) living condition; (e) insurance access and coverage]	*n* = 4^12^, ^42^, ^48^, ^51^
Patient's lifestyle factors [(a) substance abuse; (b) social situations; (c) physical activities]	*n* = 2^42^, ^50^
*Healthcare system related*	
Related to the patient–HCP relationship [(a) communication abilities; (b) trust in provider; (c) provision of patient education, training, and follow‐up; (d) time availability of consultation]	0
Directly related to the different figures and institutions involved in healthcare [(a) access of barriers to the system; (b) quality and cost of health services; (c) insurance coverage and co‐payment; (d) provider continuity; (e) drug supply; (f) regulation process; (g) provision of feedback and training to HCP; (h) community support available to patients]	*n* = 1^40^
*Disease related*	
(a) progress, duration, and severity of the condition and its symptomatology; (b) level of disability caused by the condition at the physical, psychological, social, and vocational levels; (c) existence of co‐morbidities	*n* = 3^32^, ^42^, ^50^
*Treatment related*	
Treatment regimen [(a) complexity and duration of the treatment; (b) patient friendliness of the regimen; (c) interference in the routine of the patient; (d) variation and changes of the treatment]	*n* = 2^32^, ^51^
Treatment effects [(a) appearance of the beneficial effects or side effects; (b) experience of failures in previous treatments]	0
Treatment properties [(a) formulation and physical properties of medication; (b) cost of treatment]	0
*Patient related*	
Unalterable characteristics [(a) demographics; (b) experience with treatment and treatment setting; (c) physical characteristics of the patient]	*n* = 5^12^, ^32^, ^48^, ^50^, ^51^
Cognitive and psychological factors [(a) health literacy; (b) perceptions, beliefs, and concerns of the patients regarding their condition; (c) motivation and ability to manage the condition]	*n* = 1^48^
Behavioral factors [(a) lifestyle of the patient; (b) organization; (c) planning abilities]	*n* = 1^50^

## 4 DISCUSSION

In this study, we describe the state of the art of the existing scientific experimental evidence on the factors and determinants that influence patients' adherence to treatment. As can be seen in the results section and the Supplementary Material, many studies have examined the effects of several factors and determinants on adherence to treatment. In particular regarding socioeconomic factors, most studies have considered determinants from the patients' background or environment, including their financial status, education, employment status, and living condition. Besides that significant associations have been found, these factors and determinants are difficult to modify or influence. Other factors studied relate to the existence of social support networks, which have been found to significantly affect adherence to treatment by several studies.^13^, ^14^, ^15^ Easily modifiable patient lifestyle factors have also been identified to have a significant contribution to adherence levels.^18^, ^21^, ^22^, ^24^, ^25^ Multiple studies have explored the effect of those factors on adherence related to the patient–HCP relationship and to the different figures and institutions involved in healthcare. From these, the most relevant factor associated with adherence to treatment is the provision of education and follow‐up to patients. In fact, several studies have identified its effect on the level of adherence to treatment,^23^, ^26^, ^27^, ^28^, ^29^, ^30^, ^31^, ^32^, ^33^, ^34^, ^35^ although some studies did not find any significant relation. This evidence is highly relevant as it can guide future interventions and guidelines that can help improve patients' adherence to treatment.

The characteristics of the treatment‐related factors, the duration, and its symptomatology have also been identified as having a strong influence on the patients' adherence levels. Furthermore, not only the condition but also the treatment characteristics have been identified as strong influencers, with complexity and duration of the treatment being the major factors.^19^, ^21^, ^33^, ^42^, ^43^, ^44^, ^46^ The relevance of identifying treatment complexity as an adherence determinant may help HCPs when deciding on the treatment options available for a specific patient and the associated risk of non‐adherence to such treatment.

Finally, there are some factors that are related to the physical and behavioral characteristics of the patients and their environment. Factors like age, gender, and ethnicity that are unalterable for the treatment purpose have been identified by many studies as being associated with treatment adherence. However, not all studies agree on the direction of the effect of these factors, which indicates that the effects of these factors can be highly dependent on the study setting (e.g., type of disease, type of treatment, intervention, participants included). Other factors identified as modifiers of the adherence levels were factors related to the patients' health literacy and pre‐existing beliefs and concerns about the condition and the treatment (outcomes).^14^, ^16^, ^18^, ^20^, ^24^, ^39^, ^47^, ^49^ Other relevant factors are those related to the patients' lifestyle, their self‐efficacy and planning abilities.^18^, ^24^, ^34^, ^43^, ^44^, ^47^, ^50^ The identification of these factors related to the competences of the patients, their behaviors, and psychosocial factors is highly relevant to better understand a patient's behavior toward recommended treatments and to better design approaches to improve the patient's adherence levels.

It should be noted, however, that this study has some limitations. First, the eligibility criteria limited the search to those studies published in the last decade in English. Still, most studies nowadays are published in English, and we see the studies do not show a bias toward studies based on English‐speaking regions. Second, our SR has shown that regarding adherence to treatment, most studies focus on adherence to medication and do not include additional treatment options, such as lifestyle changes, which are necessary in most cases. Subsequently, we also see that most of the studies rely only on self‐reported data (*N* = 31), a small number of studies used pill counts (*N* = 7) or devices on medication (*N* = 7), and only two used biochemical analytic data. Furthermore, most studies have used only one type of adherence measurement, making it difficult to compare the outcomes. Especially considering that differences in measuring methodology may lead to differences in the assessment of adherence levels. Importantly, the fact that self‐reported data carries the biases of recall and social desirability, along with its lack of granularity and general overestimation of adherence, is a limitation for the accuracy and precision of the data collected. Third, none of the studies have included patient adherence to treatment across the most common diseases (e.g., cardiovascular, oncology, immunology, neurology, endocrinology, and rare disease), making a comprehensive understanding of patient adherence difficult. In fact, only a limited number of the included studies covered multiple of these condition areas, and most focused only on one area. Another important lesson learned is that most of the studies consider participants from one country only, which makes it challenging to assess generalizability of the obtained results to other countries or regions where socioeconomic and healthcare system‐related factors might significantly differ. Remarkably, none of the studies included the cost of treatment in their analyses, although this is an important determinant of adherence to treatment, considering the importance of the socioeconomic factors in selected studies. Fourth, regarding the review process, having such a broad topic and scope (including several kinds of conditions, treatments, measures of adherence, etc.) challenges the proper feeding of the ASReview tool. This limitation has been overcome by performing several additional training rounds before getting the final prioritization algorithm. Fifth, most of the studies included do not consider the heterogeneity of treatment effect at local level, whereas most of the modeling in the selected studies carried out mainly through standard regression techniques use ‘average models’ that do not allow full identification of the local heterogeneity of treatments of effects identifiable in subgroups of individuals according to contemporary statistical methods. The identification of heterogeneous treatment effects is at the basis of personalized treatments and influence adherence to the treatment significantly, something already in other, not health‐related domains. Sixth, we did not report the average goodness of fitness of these studies in their explanation of patients' adherence to treatments. Future research could analyze the studies in more detail to provide the goodness of fitness of the selected studies to have a more in‐depth interpretation of the adherence outcomes. Lastly, most of the literature studying factors influencing adherence to treatment relies on patient self‐reported data, which, as discussed above, carries its own biases. These are vital lessons learned for future steps in scientific research in patient adherence to treatment.

## 5 CONCLUSION

A better understanding of patients' adherence to treatment is important to maximize the benefit of healthcare provided to patients, in order to improve health‐related outcomes and reduce costs. The results of this SR show that a large number of studies show the effects and associations of several factors and determinants on adherence to treatment. This study analyzed the reported effects of factors related to the patient's characteristics and behaviors, the characteristics of the condition and its treatment, as well as characteristics of the healthcare system and socioeconomic environment. Despite this overview of available data on the scientific literature presented in this document, it is highly relevant to conduct more scientific research using high quality standards (e.g., randomized controlled trials, across disease areas, longitudinal) in patient adherence to maximize the benefit of healthcare provision for patients, which is a key factor for various subsequent health outcomes.

## AUTHOR CONTRIBUTIONS

FF, ARUW, KvH, and FLV have conducted the systematic review and analyzed the majority of the papers. All authors have analyzed part of the articles included in the SR and have read and approved the final manuscript.

## CONFLICT OF INTEREST STATEMENT

FF, ARUW, KvH, ARL, JIC, ET, AM, JBC, BM, PA, GF, LM, JC, RS, EC, EK, NB, GB, KG, KS, LDR, FLV have no conflict of, or competing, interest.

## FUNDING INFORMATION

We acknowledge funding by the IMI funded BEAMER project, contract 101034369. IMI is funded by the European Union Horizon 2020, EFPIA and associated partners – Link2Trials.

## ETHICS APPROVAL AND CONSENT TO PARTICIPATE

Not applicable.

## CONSENT FOR PUBLICATION

Not applicable.

## Data Availability

The current study does not contain any personal or individual data.
